# Effects of high-intensity interval training versus continuous aerobic training on body weight, body mass index, and body fat percentage in youth with overweight or obesity: a systematic review and meta-analysis

**DOI:** 10.3389/fped.2026.1678900

**Published:** 2026-09-25

**Authors:** Yuan Jin, Yuanli Zhou, Huaxing Zhang

**Affiliations:** 1 People’s Sports Publishing House, Beijing, China; 2 Chongqing Agricultural Mechanization School, Chongqing, China; 3 City University Malaysia, Petaling Jaya, Selangor, Malaysia

**Keywords:** high-intensity interval training, continuous aerobic training, overweight, obesity, youth, body composition, systematic review, meta-analysis

## Abstract

**Background:**

Overweight and obesity in youth are associated with substantial short- and long-term health burdens, and structured exercise is a central component of management. Whether HIIT offers greater improvements in body composition than continuous aerobic training remains uncertain.

**Methods:**

This systematic review and meta-analysis followed PRISMA 2020. PubMed, Embase, Web of Science, CNKI, Wanfang, and VIP were searched from inception to December 31, 2024 for randomized trials directly comparing high-intensity interval-based training, including high-intensity interval exercise and sprint-interval protocols, with continuous aerobic training in youth with overweight or obesity. Outcomes were body weight, BMI, and body fat percentage. Risk of bias was assessed with RoB 2. Random-effects models with REML estimation and modified Hartung–Knapp inference generated mean differences (MDs) and 95% confidence intervals (CIs). Sensitivity, subgroup, and small-study-effect analyses were also performed.

**Results:**

Fourteen independent randomized trials were included. For body weight, 13 trials involving 409 participants showed no statistically significant difference between HIIT and continuous aerobic training (MD = −0.37 kg, 95% CI −0.89 to 0.15; *P* = 0.146; *I*^2^ = 0%). For BMI, 14 trials involving 432 participants also showed no significant difference (MD = −0.14 kg/m^2^, 95% CI −0.31 to 0.02; *P* = 0.089; *I*^2^ = 0%). For body fat percentage, 11 trials involving 331 participants showed no significant difference (MD = −0.33 percentage points, 95% CI −1.33 to 0.67; *P* = 0.481), with moderate-to-substantial heterogeneity (*I*^2^ = 60.3%). Sensitivity analyses did not materially alter the findings, and subgroup analyses did not identify reliable effect modification by weekly frequency, intervention duration, or mean age. Egger’s tests did not detect statistically significant small-study effects. One trial was at low overall risk of bias, whereas 13 raised some concerns.

**Conclusion:**

Randomized evidence identified through December 31, 2024 does not show that HIIT provides greater reductions in body weight, BMI, or body fat percentage than continuous aerobic training. HIIT may be considered an alternative exercise format, but the limited evidence base and heterogeneity in body fat outcomes warrant cautious interpretation.

## 1 Introduction

Childhood and adolescent obesity is a substantial global health problem. A 2024 meta-analysis of 2,033 studies from 154 countries estimated that 8.5% of children and adolescents had obesity and 22.2% had excess weight, with prevalence increasing over time (1). China has undergone a marked nutritional transition and now carries a large absolute burden of childhood obesity. Although national and multisectoral policies have expanded, their implementation and coordination remain challenging (2). Obesity during growth is associated with hypertension, insulin resistance, dyslipidemia, fatty liver disease, psychosocial difficulties, and an increased likelihood of obesity persisting into adulthood (3). These consequences extend beyond body size and may affect health, functioning, and quality of life across the life course. Because childhood and adolescence are periods of rapid physical and behavioral development, prevention and treatment need to improve health while supporting normal growth and avoiding stigmatizing approaches.

Management of obesity in children and adolescents is therefore generally multicomponent, integrating nutrition, behavioral support, family involvement, and physical activity. Current clinical guidance emphasizes intensive, family-centered health behavior and lifestyle treatment, while pharmacological or surgical treatment may be considered for selected adolescents with severe obesity under specialist care (4). Appropriately prescribed and supervised exercise is a central component of lifestyle management because it can be adapted to age, functional capacity, and treatment goals. A recent meta-analysis of 113 randomized trials involving 8,514 children and adolescents with overweight or obesity found that exercise interventions improved BMI, waist circumference, and body fat percentage, although the magnitude of benefit varied across programs and settings (5). Exercise should therefore be viewed as part of comprehensive care rather than as a stand-alone guarantee of weight normalization, particularly when intervention duration, training dose, dietary intake, and adherence differ.

Continuous aerobic training involves sustained exercise performed at a relatively stable intensity without planned high-intensity intervals. By contrast, high-intensity interval training (HIIT) uses repeated bouts of vigorous or near-maximal exercise interspersed with active or passive recovery; in this review, the term also encompasses high-intensity interval exercise and sprint-interval protocols. HIIT has attracted interest because it can be delivered through different exercise modalities and may achieve meaningful cardiorespiratory stimulation within relatively short sessions (6). In youth with overweight or obesity, earlier evidence suggested that HIIT could improve aerobic fitness and selected cardiometabolic outcomes, but was insufficient to establish that it was more effective than continuous endurance training for body composition (7). A meta-analysis restricted to randomized comparisons of HIIT and moderate-intensity continuous training found no significant differences in body weight, BMI, or body fat percentage, although HIIT produced larger improvements in cardiorespiratory fitness and systolic blood pressure (8). Similarly, a randomized trial in children with obesity reported a greater fitness response to HIIT without a corresponding advantage in whole-body or regional adiposity (9).

The relative effects of HIIT and continuous aerobic training on body composition therefore remain uncertain. Recent direct-comparison evidence has continued to show broadly similar effects for body weight and BMI, while estimates for body fat percentage remain sensitive to the studies and analytical methods used (10). These outcomes should not be treated as interchangeable. Body weight is influenced by growth and changes in both fat and fat-free mass, whereas BMI is practical for surveillance but does not directly distinguish adipose tissue from lean tissue; different pediatric BMI metrics also vary in their association with measured adiposity and cardiometabolic risk (11). Body fat percentage more directly reflects the proportion of fat mass, but estimates can differ according to the measurement method, and the accuracy of dual-energy x-ray absorptiometry may be affected by tissue mass and pubertal status (12). Accordingly, this systematic review and meta-analysis compared the effects of high-intensity interval-based training and continuous aerobic training on body weight, BMI, and body fat percentage in youth with overweight or obesity. We also explored whether participant age, intervention duration, and weekly training frequency modified the observed effects.

## 2 Methods

### 2.1 Reporting guidelines

This systematic review and meta-analysis was reported in accordance with the PRISMA 2020 Statement (13). The processes of study identification, screening, eligibility assessment, and inclusion were summarized in a PRISMA flow diagram.

### 2.2 Search strategy

PubMed, Embase, Web of Science, CNKI, Wanfang, and VIP were searched from database inception to December 31, 2024 to identify potentially eligible studies comparing high-intensity interval-based training with continuous aerobic training in children and adolescents with overweight or obesity. Controlled vocabulary and free-text terms related to “high-intensity interval training,” “high-intensity interval exercise,” “sprint interval training,” “interval training,” “continuous aerobic training,” “moderate-intensity continuous training,” “endurance training,” “overweight,” “obesity,” “children,” “adolescents,” and “youth” were combined using the Boolean operators “AND” and “OR”. Equivalent Chinese terms were used in the Chinese databases. Searches were limited to English- and Chinese-language publications. Reference lists of included studies and relevant reviews were also manually screened to identify additional eligible reports.

### 2.3 Eligibility criteria

Studies were eligible if they: (1) were randomized controlled trials; (2) included participants aged 6–19 years with overweight or obesity according to defined criteria; (3) directly compared high-intensity interval-based training, including HIIT, HIIE, or sprint interval training, with continuous aerobic training; (4) reported at least one outcome of interest—body weight, BMI, or body fat percentage; and (5) were full-text articles published in English or Chinese with sufficient data for effect-size calculation. Studies with dietary, educational, or lifestyle co-interventions were eligible only when the same co-intervention was provided to both groups.

Studies were excluded if they: (1) were non-randomized, single-arm, crossover, or acute single-session studies; (2) did not directly compare eligible interval and continuous training programs; (3) involved unequal dietary, pharmacological, or other co-interventions between groups; (4) lacked sufficient data for analysis; or (5) were reviews, commentaries, protocols, conference abstracts, or meta-analyses. Baseline differences between groups were not used as an exclusion criterion. Studies in which random allocation was not clearly reported were excluded from the primary analysis but were considered in sensitivity analyses when all other eligibility criteria were met. Study eligibility decisions were made by the author team and resolved by consensus.

### 2.4 Study selection and data extraction

All records were imported into EndNote X20, and duplicates were removed. Study screening was conducted by the author team, first by title and abstract and then by full-text assessment against the eligibility criteria; disagreements were resolved by consensus.

Using a standardized Excel form, the author team extracted study characteristics, participant characteristics, intervention and comparator details, co-interventions, outcome measures, and group-level data for body weight, BMI, and body fat percentage. Adjusted between-group estimates were used when available; otherwise, change scores or post-intervention values were extracted. Multiple reports from the same trial were linked and treated as a single study, and eligible arms in multi-arm trials were combined to avoid double-counting participants (14). When necessary, standard errors were derived from reported confidence intervals, *P* values, or other test statistics. For one included trial, the standard errors for body weight and BMI were reconstructed from the reported group-by-time F statistics because exact change-score variances were unavailable.

### 2.5 Risk of bias assessment

Risk of bias for the outcomes included in the meta-analysis was assessed using the Cochrane Risk of Bias 2 (RoB 2) tool (15). Five domains were evaluated: bias arising from the randomization process, bias due to deviations from intended interventions, bias due to missing outcome data, bias in measurement of the outcome, and bias in selection of the reported result. Each domain and the overall risk of bias were rated as low risk, some concerns, or high risk. Risk-of-bias assessments were conducted by the author team and resolved by consensus. The results were summarized in tabular format.

### 2.6 Data synthesis and statistical analysis

Meta-analyses were performed using Stata/SE version 15.1 (StataCorp, College Station, TX, USA). Body weight, body mass index (BMI), and body fat percentage were pooled as mean differences (MDs) with 95% confidence intervals (CIs). Effects were calculated as high-intensity interval-based training minus continuous aerobic training; therefore, negative values favored interval training. Eligible arms in multi-arm trials were combined to avoid double-counting participants (14).

Random-effects models with restricted maximum likelihood estimation (16) and modified Hartung–Knapp inference (17) were used for all outcomes. Heterogeneity was assessed using Cochran's *Q*, *τ*^2^, and *I*^2^ statistics, with two-sided *P* < 0.05 considered statistically significant. Robustness was assessed using leave-one-out analyses and sensitivity analyses excluding the reconstructed Su effects and including the Leite study, for which random allocation was not clearly reported. Exploratory subgroup analyses were conducted by weekly training frequency (≤3 vs. ≥4 sessions/week), intervention duration (<12 vs. ≥12 weeks), and mean participant age (<13 vs. ≥13 years), with differences assessed using interaction tests. Univariable meta-regression separately examined publication year, mean age, intervention duration, HIIT frequency, sex composition, and structured co-intervention; these analyses were exploratory and were not adjusted for multiple testing (18). Small-study effects were assessed using funnel plots and Egger's regression test in the primary analyses (19).

## 3 Results

### 3.1 Literature search and selection

The database search identified 792 records. After removing 265 duplicates, 527 records underwent title and abstract screening, of which 468 were excluded. Fifty-nine full-text reports were sought for retrieval; two could not be obtained, leaving 57 reports for eligibility assessment. Forty-three reports were excluded because of an ineligible comparator (*n* = 14), a non-randomized or otherwise inappropriate design (*n* = 10), an ineligible population (*n* = 7), unavailable outcome data (*n* = 6), or a duplicate cohort or secondary report (*n* = 6). Ultimately, 14 independent randomized trials were included in the meta-analysis (Figure 1).

**Figure 1:**
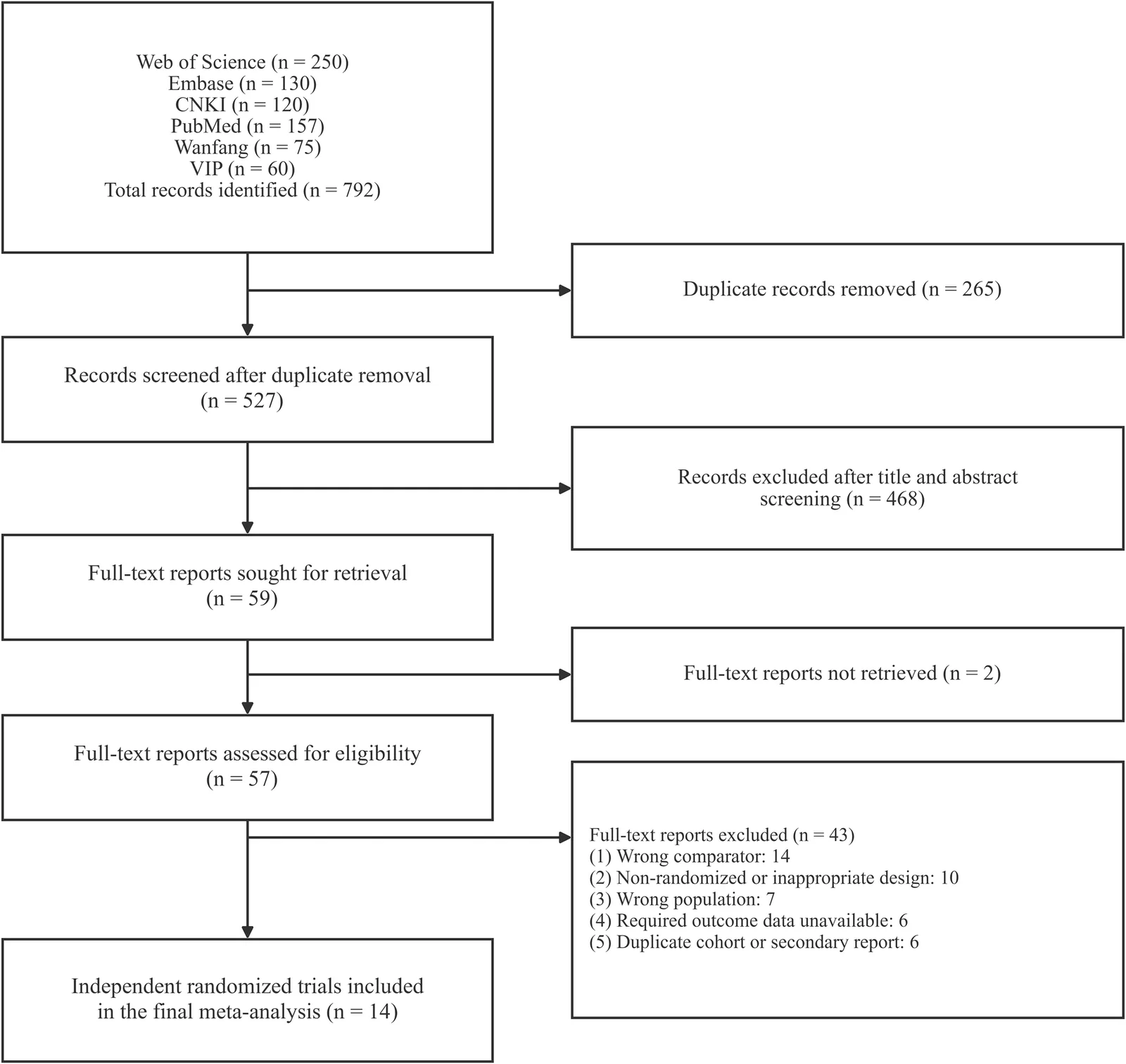
PRISMA 2020 flow diagram of study identification, screening, eligibility assessment, and inclusion.

### 3.2 Characteristics of included studies

Fourteen independent randomized trials published between 2004 and 2024 were included (Table 1), including up to 432 participants with overweight or obesity. The studies were conducted in Brazil, Tunisia, the United States, Iran, Italy, Australia and Norway, China, and France. Reported participant ages ranged from approximately 7 to 19 years; nine trials enrolled boys only, whereas five included both boys and girls. Thirteen trials reported body weight, all 14 reported BMI, and 11 reported body fat percentage, contributing 409, 432, and 331 participants to the respective analyses.

**Table 1:** Characteristics of included randomized trials comparing high-intensity interval-based training with continuous aerobic training in youth with overweight or obesity.

Study; country	Participants	Analyzed, *n* (interval/CAT)	Interval intervention	Continuous comparator	Training dose	Co-intervention	Outcomes
Fernandez et al. (2004); Brazil (20)	Boys with severe obesity; 15–19 y (mean 16.08)	HIIT 10/CAT 9	Cycle sprints: 11–14 × 30–45 s near maximal effort; 3-min active recovery	Continuous cycling at ventilatory threshold; 40–60 min	3/wk; 12 wk	Nutritional guidance in all arms	Body weight; BMI; Body fat percentage
Corte de Araujo et al. (2012); Brazil (21)	Children with obesity; boys and girls; 8–12 y	HIT 15/ET 15	Treadmill HIT: 3–6 × 60 s at 100% peak velocity; 3-min recovery at 50% velocity	Continuous treadmill exercise at 80% peak HR; 30–60 min	2/wk; 12 wk	Healthy-eating counselling	Body weight; BMI; Body fat percentage
Koubaa et al. (2013); Tunisia (22)	Boys with obesity; mean 13.0/12.9 y	IT 14/CT 15	Intermittent running: 2-min bouts at 80% vVO_2_max; 1-min recovery	Continuous running at 60%–70% vVO_2_max; 30–40 min	3/wk; 12 wk	Not reported	Body weight; BMI
Starkoff et al. (2014); USA (23)	Sedentary adolescents with obesity; mean 14.7 y; 17 girls/10 boys	HIIE 14/CAT 13	Cycling HIIE: 10 × 2 min at 90%–95% age-predicted HRmax; 1-min active recovery	Continuous cycling at 65%–70% age-predicted HRmax; 30 min	3/wk; 6 wk	None reported	Body weight; BMI; Body fat percentage
Kargarfard et al. (2016); Iran (24)	Boys aged 10–14 y; obesity stratum used	HIIT 10/ET 10	Progressive treadmill intervals at 60%–90% HR reserve; 4-min bouts with 2-min recovery	Continuous treadmill endurance exercise at 60%–95% HR reserve	HIIT 3/wk; ET 5/wk; 8 wk	None reported	Body weight; BMI
Lazzer et al. (2017); Italy (25)	Hospitalized boys with severe obesity; 15–17 y	HIIT 10/CAT 20^a^	Repeated 40-s walking bouts at 100% VO_2_max; 5 min at 40% VO_2_max	Two continuous arms pooled: LI at 40% and HI at 70% VO_2_max	10/wk; 3 wk	Energy restriction, nutrition education and psychological counselling	Body weight; BMI; Body fat percentage
Dias et al. (2018); Australia/Norway (9)	Children with obesity; boys and girls; 7–16 y	23/25 for weight/BMI; 18/22 for body fat^a^	HIIT: 4 × 4 min at 85%–95% HRmax; 3-min active recovery	MICT at 60%–70% HRmax; 44 min	3/wk; 12 wk	Nutrition advice in all arms	Body weight; BMI; Body fat percentage
Liang and Hao (2018); China (26)	Boys with simple obesity; school age	HIIT 9/MICT 9	Treadmill HIIT: 3–6 × 60 s at maximal speed; 3-min recovery at 50% speed	Continuous treadmill exercise at 80% HRpeak; 30–60 min	3/wk; 12 wk	None stated	Body weight; BMI; Body fat percentage
Miguet et al. (2020); France (27)	Adolescents with obesity; 11–15 y; 72% girls	HIIT 22/MICT 21	Cycle HIIT: 30 s work/30 s active recovery for 15 min; 75%–90% VO2peak	Continuous cycling at 60% VO2peak; 45 min	2/wk; 16 wk	Common diet, aquatic/leisure activity, strength training and education	Body weight; BMI; Body fat percentage
Paahoo et al. (2020); Iran (28)	Boys with overweight or obesity; mean 11.06 y	HIIT 15/CAT 15	Running HIIT at 100%–110% maximal aerobic speed	Continuous aerobic training at 40%–70% HR reserve	3/wk; 12 wk	None reported	Body weight; BMI; Body fat percentage
Cao et al. (2022); China (29)	Boys with obesity; 10–13 y	HIIT 12/MICT 11	Running HIIT: 2 sets of 8 × 15 s at 90%–100% MAS; 15 s at 50% MAS	Continuous running at 60%–70% MAS; 30 min	3/wk; 12 wk	None reported	BMI; Body fat percentage
Li et al. (2023); China (30)	Children with obesity; 10–13 y; boys and girls	HIIT 16/MICT 15	Running HIIT: 2 sets of 8 × 15 s at 100%–120% MAS; 15 s at 50% MAS	Continuous running at 60%–80% MAS; 20–40 min	3/wk; 12 wk	Free diet monitored by dietary recall	Body weight; BMI; Body fat percentage
Zuo et al. (2023); China (31)	Boys with overweight; 7–10 y	HIIE 20/MICE 20	Running HIIE: 2 sets of 15 × 20 s at 85%–95% MAS; 20 s at 50% MAS	Continuous running at 60%–70% MAS; 30 min	3/wk; 8 wk	Regular physical education continued	Body weight; BMI; Body fat percentage
Su et al. (2024); China (32)	Boys with obesity in a weight-loss camp; mean 14/15 y	HIIT 22/MICT 22 (43 completed)^a^	Treadmill HIIT: 10 × 1 min at 85%–95% peak HR; 2 min at 60%–70% peak HR	Continuous treadmill exercise at 65%–75% peak HR; 35 min	3/wk; 8 wk	Dietary health talks and energy-intake recommendations	Body weight; BMI

CAT, continuous aerobic training; CT, continuous training; ET, endurance training; HIIE, high-intensity interval exercise; HIIT/HIT, high-intensity interval training; HR, heart rate; HRmax, maximal heart rate; HRR, heart-rate reserve; LI/HI, low-/high-intensity continuous training; MAS, maximal aerobic speed; MICE/MICT, moderate-intensity continuous exercise/training; vVO_2_max, velocity at maximal oxygen uptake.

a Lazzer et al.: the two eligible continuous-training arms were pooled. Dias et al.: available-case sample sizes varied by outcome. Su et al.: 44 participants were randomized and 43 completed the intervention.

The high-intensity interval-based interventions included cycling sprints, treadmill intervals, intermittent running, and repeated high-intensity walking, whereas the comparators consisted of continuous cycling, treadmill exercise, running, or other endurance training. Interventions lasted 3–16 weeks and were performed 2–10 times per week, with 12-week programs being the most common. Exercise intensity was prescribed using heart rate, heart-rate reserve, maximal aerobic speed, oxygen-uptake-related measures, or ventilatory threshold. Several trials also provided comparable nutritional, educational, or lifestyle co-interventions to both groups. Detailed study characteristics are presented in Table 1.

### 3.3 Risk of bias assessment

Risk-of-bias assessments for the included trials are presented in Supplementary Table S1. Of the 14 trials, one (7.1%) was judged to be at low risk of bias overall, 13 (92.9%) raised some concerns, and none was judged to be at high risk. The most frequent concerns involved the randomization process in 10 trials (71.4%), mainly because the allocation process was incompletely reported, and missing outcome data in six trials (42.9%), primarily because of attrition and complete-case analyses. All trials were judged to be at low risk for deviations from intended interventions, outcome measurement, and selection of the reported result.

### 3.4 Effects on body-composition outcomes

All effect estimates were calculated as HIIT minus continuous aerobic training; therefore, negative mean differences favored HIIT. The pooled results are presented in Figure 2, and detailed estimates are provided in Table 2.

**Figure 2:**
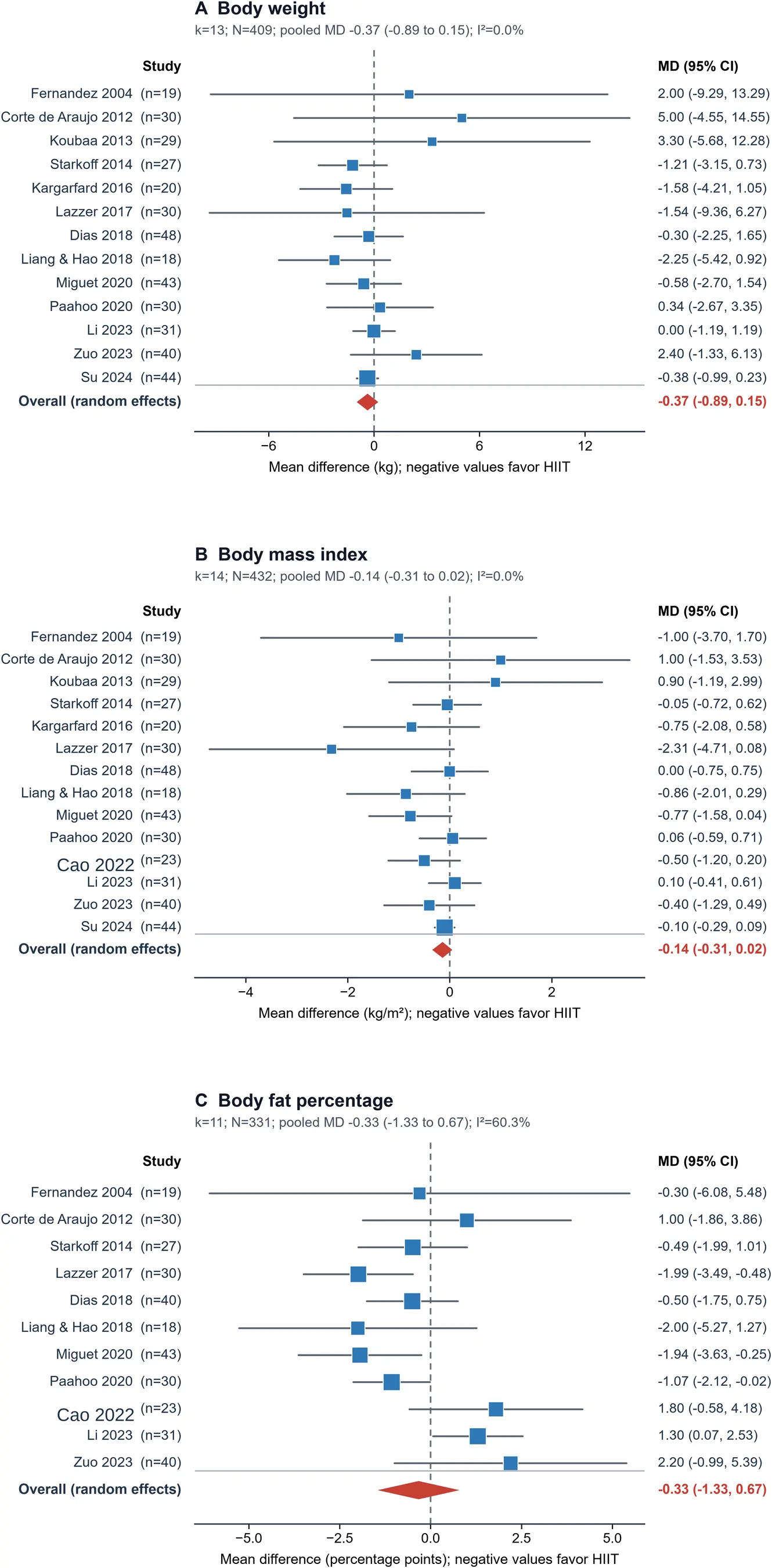
Forest plots of the effects of high-intensity interval-based training versus continuous aerobic training on **(A)** body weight, **(B)** body mass index, and **(C)** body fat percentage.

**Table 2:** Meta-analysis and sensitivity analyses of body-composition outcomes.

Analysis	Outcome	Studies, *k*	Participants, *N*	MD (95% CI)	*P* value	*τ* ^2^	*I*^2^ (%)	*Q*-test *P*	Egger *P*
Primary analysis	Body weight (kg)	13	409	−0.37 (−0.89 to 0.15)	0.146	0.000	0.0	0.805	0.475
Primary analysis	BMI (kg/m^2^)	14	432	−0.14 (−0.31 to 0.02)	0.089	0.000	0.0	0.458	0.213
Primary analysis	Body fat (percentage points)	11	331	−0.33 (−1.33 to 0.67)	0.481	1.166	60.3	0.008	0.570
Sensitivity: excluding reconstructed Su effects	Body weight (kg)	12	365	−0.36 (−1.17 to 0.45)	0.354	0.000	0.0	0.736	–
Sensitivity: excluding reconstructed Su effects	BMI (kg/m^2^)	13	388	−0.21 (−0.48 to 0.07)	0.125	0.000	0.0	0.414	–
Sensitivity: including Leite (allocation unclear)	Body weight (kg)	14	449	−0.36 (−0.87 to 0.15)	0.153	0.000	0.0	0.820	–
Sensitivity: including Leite (allocation unclear)	BMI (kg/m^2^)	15	472	−0.13 (−0.31 to 0.05)	0.140	0.000	0.0	0.298	–
Sensitivity: including Leite (allocation unclear)	Body fat (percentage points)	12	371	−0.18 (−1.18 to 0.81)	0.695	1.295	61.4	0.005	–

Random-effects models used restricted maximum likelihood estimation and modified Hartung–Knapp inference. MD was calculated as high-intensity interval-based training minus continuous aerobic training; negative values favor HIIT. The Su sensitivity analysis excluded reconstructed body weight and BMI effects. The Leite sensitivity analysis included a direct-comparison study for which random allocation was not clearly reported. Egger’s test was applied only to the primary analyses.

#### 3.4.1 Body weight

Thirteen trials involving 409 participants reported body weight. The pooled estimate slightly favored HIIT, but the difference between HIIT and continuous aerobic training was not statistically significant (MD = −0.37 kg, 95% CI −0.89 to 0.15; *P* = 0.146). No between-study heterogeneity was detected (*τ*^2^ = 0.000; *I*^2^ = 0%; Cochran's *Q* = 7.74, *P* = 0.805), indicating statistically consistent findings across the included trials.

#### 3.4.2 Body mass index

Fourteen trials involving 432 participants contributed data for BMI. The pooled estimate showed no statistically significant difference between HIIT and continuous aerobic training (MD = −0.14 kg/m^2^, 95% CI −0.31 to 0.02; *P* = 0.089). Between-study heterogeneity was not detected (*τ*^2^ = 0.000; *I*^2^ = 0%; Cochran's *Q* = 12.86, *P* = 0.458).

#### 3.4.3 Body fat percentage

Eleven trials involving 331 participants reported body fat percentage. Although the pooled estimate favored HIIT, the difference was not statistically significant (MD = −0.33 percentage points, 95% CI −1.33 to 0.67; *P* = 0.481). Moderate-to-substantial heterogeneity was observed (*τ*^2^ = 1.166; *I*^2^ = 60.3%; Cochran's *Q* = 24.02, *P* = 0.008), indicating variability in the effects reported across trials and warranting cautious interpretation of the pooled estimate.

### 3.5 Sensitivity analyses

Leave-one-out analyses showed that omitting any individual trial did not materially alter the pooled estimates, and all corresponding 95% CIs continued to include zero. After excluding the reconstructed body weight and BMI effects from Su et al. (32), the results remained non-significant for body weight (MD = −0.36 kg, 95% CI −1.17 to 0.45; *P* = 0.354) and BMI (MD = −0.21 kg/m^2^, 95% CI −0.48 to 0.07; *P* = 0.125). Including the direct-comparison study by Leite et al. (33), in which random allocation was unclear, also did not change the conclusions for body weight (MD = −0.36 kg, 95% CI −0.87 to 0.15), BMI (MD = −0.13 kg/m^2^, 95% CI −0.31 to 0.05), or body fat percentage (MD = −0.18 percentage points, 95% CI −1.18 to 0.81). These findings indicated that the primary results were robust to the sensitivity analyses. Detailed primary and sensitivity-analysis estimates are presented in Table 2.

### 3.6 Small-study effects

Funnel plots for body weight, BMI, and body fat percentage are presented in Figure 3. Egger's regression tests did not detect statistically significant small-study effects for body weight (*P* = 0.475), BMI (*P* = 0.213), or body fat percentage (*P* = 0.570). However, because only 11–14 trials were available for each outcome, these findings should be interpreted cautiously.

**Figure 3:**
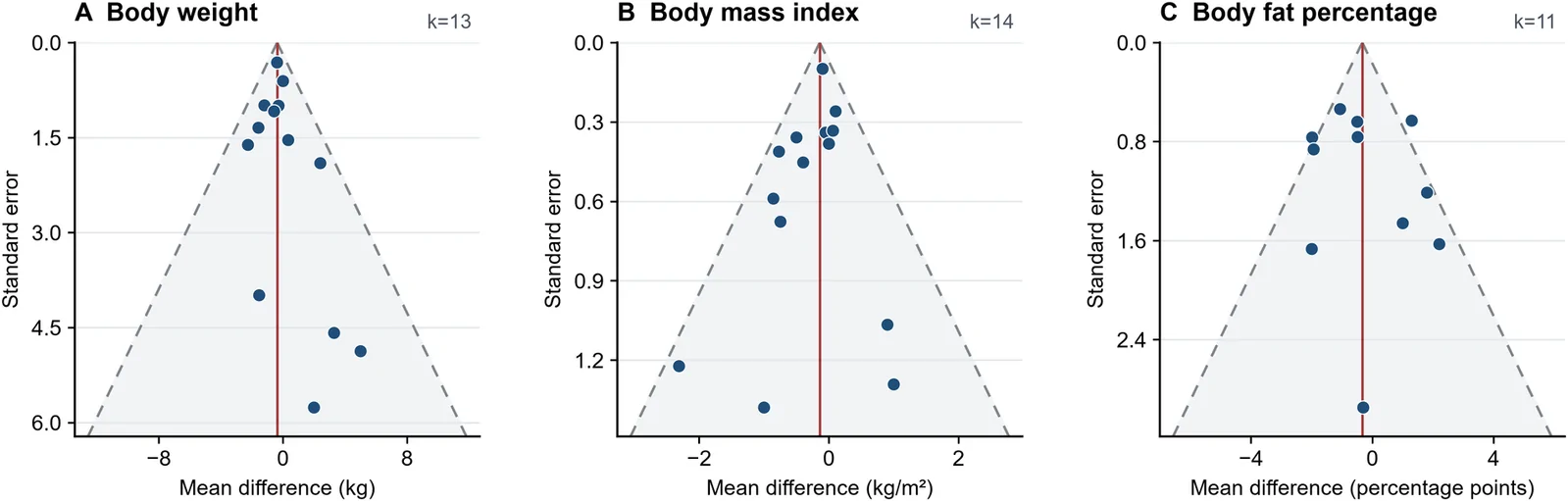
Funnel plots for the assessment of small-study effects for **(A)** body weight, **(B)** body mass index, and **(C)** body fat percentage.

### 3.7 Meta-regression analysis

Univariable meta-regression analyses were performed to explore whether publication year, mean age, intervention duration, HIIT frequency, sex composition, or structured co-intervention modified the effects of HIIT on body-composition outcomes. For body weight and BMI, none of the examined moderators showed a statistically significant association with the pooled effect estimates. For body fat percentage, mean age was associated with the intervention effect (*β* = −0.449, 95% CI −0.872 to −0.026; *P* = 0.040), suggesting that studies with older participants tended to show larger effects favoring HIIT. Other moderators, including publication year, intervention duration, HIIT frequency, sex composition, and structured co-intervention, were not statistically significant for body fat percentage. Full meta-regression results are presented in Table 3.

**Table 3:** Univariable meta-regression analyses of potential moderators of high-intensity interval-based training effects on body-composition outcomes.

Outcome	Moderator	Unit/comparison	Studies, *k*	*β* (95% CI)	*P* value	Residual *τ*^2^	R^2^ analog (%)
Body weight (kg)	Publication year	per 5-year increase	13	0.286 (−0.544 to 1.115)	0.465	0.000	–
	Mean age	per 1-year increase	12	−0.199 (−0.537 to 0.139)	0.219	0.000	–
	Intervention duration	per 4-week increase	13	0.197 (−0.665 to 1.058)	0.626	0.000	–
	HIIT frequency	per 1-session/week increase	13	−0.142 (−1.248 to 0.963)	0.782	0.000	–
	Sex composition	mixed-sex vs. boys only	13	0.067 (−1.057 to 1.191)	0.898	0.000	–
	Structured co-intervention	yes vs. no/not reported	13	0.003 (−1.133 to 1.140)	0.995	0.000	–
BMI (kg/m^2^)	Publication year	per 5-year increase	14	0.066 (−0.214 to 0.347)	0.616	0.000	–
	Mean age	per 1-year increase	13	−0.005 (−0.107 to 0.097)	0.917	0.000	–
	Intervention duration	per 4-week increase	14	−0.060 (−0.361 to 0.241)	0.672	0.000	–
	HIIT frequency	per 1-session/week increase	14	−0.182 (−0.530 to 0.165)	0.275	0.000	–
	Sex composition	mixed-sex vs. boys only	14	0.100 (−0.336 to 0.536)	0.626	0.005	–
	Structured co-intervention	yes vs. no/not reported	14	0.009 (−0.369 to 0.387)	0.959	0.000	–
Body fat (percentage points)	Publication year	per 5-year increase	11	0.550 (−0.765 to 1.866)	0.369	1.065	8.7
	Mean age	per 1-year increase	10	−0.449 (−0.872 to −0.026)	0.040	0.612	50.4
	Intervention duration	per 4-week increase	11	0.170 (−0.995 to 1.336)	0.748	1.396	0.0
	HIIT frequency	per 1-session/week increase	11	−0.230 (−0.658 to 0.197)	0.254	1.085	7.0
	Sex composition	mixed-sex vs. boys only	11	0.305 (−1.838 to 2.449)	0.755	1.357	0.0
	Structured co-intervention	yes vs. no/not reported	11	−1.234 (−3.150 to 0.682)	0.179	0.917	21.4

*β* is the change in pooled MD per moderator increment or category comparison; negative estimates favor interval training. Moderators were modeled separately using REML with modified Hartung–Knapp inference. R^2^ analog is the percentage reduction in *τ*^2^ relative to the corresponding intercept-only model; – indicates zero intercept-only *τ*^2^.

### 3.8 Subgroup analysis results

Subgroup analyses were conducted according to weekly training frequency (≤3 vs. ≥4 sessions/week), intervention duration (<12 vs. ≥12 weeks), and mean participant age (<13 vs. ≥13 years). No statistically significant between-subgroup differences were observed for body weight (*P* for interaction = 0.773, 0.638, and 0.403, respectively), BMI (*P* for interaction = 0.100, 0.868, and 0.737), or body fat percentage (*P* for interaction = 0.197, 0.748, and 0.067, respectively).

The single trial in the ≥4 sessions/week subgroup reported a reduction in body fat percentage favoring HIIT (MD = −1.99 percentage points, 95% CI −3.49 to −0.48); however, the interaction test was not significant. A non-significant tendency toward a greater body-fat reduction was also observed in studies with a mean participant age of ≥13 years (MD = −1.41 percentage points, 95% CI −2.97 to 0.15) compared with those involving younger participants (MD = 0.45 percentage points, 95% CI −1.03 to 1.93; *P* for interaction = 0.067). Overall, the subgroup analyses did not identify a reliable effect modifier (Supplementary Table S2).

## 4 Discussion

This systematic review included 14 independent randomized trials comparing HIIT with continuous aerobic training in youth with overweight or obesity. HIIT did not produce a statistically significant additional reduction in body weight (MD = −0.37 kg, 95% CI −0.89 to 0.15), BMI (MD = −0.14 kg/m^2^, 95% CI −0.31 to 0.02), or body fat percentage (MD = −0.33 percentage points, 95% CI −1.33 to 0.67). Body weight and BMI results were consistent across trials, whereas the body fat analysis showed moderate-to-substantial heterogeneity (*I*^2^ = 60.3%). Leave-one-out and additional sensitivity analyses did not materially change the estimates. Egger tests did not detect statistically significant small-study effects, although their power was limited. These findings do not establish an additional body-composition benefit of HIIT over continuous aerobic training and should not be interpreted as proof of equivalence.

The overall pattern is broadly consistent with earlier syntheses. A review of HIIT in youth with overweight or obesity reported improvements relative to baseline or non-exercise conditions but found insufficient evidence that HIIT was more effective than continuous endurance training (7). A later meta-analysis restricted to direct HIIT–MICT comparisons likewise found no significant between-group differences in body weight, BMI, or body fat percentage, although HIIT produced larger gains in cardiorespiratory fitness and systolic blood pressure (8). A recent direct-comparison review also reported similar effects for most body-composition outcomes, with only an uncertain, modest signal for body fat percentage (10). Differences between pooled estimates may reflect eligibility criteria, study sets, effect measures, outcome definitions, and the handling of multi-arm or incompletely reported trials rather than a stable advantage of one training mode.

The absence of a clear difference in body weight and BMI is also compatible with individual trials. Corte de Araujo et al. found that interval and endurance training produced similar improvements in several health measures despite a substantially shorter interval-training session (21), while Dias et al. observed a larger improvement in cardiorespiratory fitness with HIIT but no corresponding advantage in whole-body or regional adiposity (9). BMI combines changes in height, fat mass, and fat-free mass, which limits its sensitivity to short-term adiposity changes during growth; different BMI metrics also show materially different associations with measured fatness in youth (11). Body weight is determined by cumulative energy balance, and greater intensity per unit time does not necessarily create a larger sustained energy deficit when total work, dietary intake, and compensatory responses differ. An exercise-dose trial in adults showed that compensation can attenuate expected weight loss and is not determined simply by training frequency or intensity (34).

The body fat result requires greater caution because its confidence interval was wide and heterogeneity was appreciable. The included trials differed in exercise mode, interval structure, intensity target, training volume, co-interventions, and body-composition assessment. Measurement differences can affect both baseline estimates and observed change: in youth with obesity, DXA showed bias relative to a four-component model, with disagreement related partly to tissue mass and pubertal status (12). Protocol variation is another plausible contributor. A systematic review of pediatric HIIT found uncertainty about the most effective combination of work duration, recovery, frequency, and program length because studies used diverse protocols and had methodological limitations (6). These sources of variation make it inappropriate to infer a general fat-loss advantage from a small number of favorable individual estimates.

The subgroup analyses did not identify a statistically reliable modifier of the effects on body weight, BMI, or body fat percentage. Interaction tests were not significant for weekly frequency, intervention duration, or mean age. The continuous-age meta-regression coefficient was statistically significant (*β* = −0.449 percentage points per year; *P* = 0.040), but the categorical age interaction was not (*P* = 0.067). Puberty is accompanied by sex-specific changes in fat mass, fat-free mass, physical capacity, and energy expenditure, so age may capture biological differences as well as differences in study design and participant characteristics (35). However, study-level meta-regression cannot establish an individual-level age response, and estimates based on few trials are vulnerable to residual confounding, multiple testing, and chance findings (18). The age signal is therefore hypothesis-generating rather than a basis for prescribing HIIT differently by age. The ≥4 sessions/week subgroup contained only one trial and cannot support a general high-frequency recommendation.

HIIT remains a reasonable alternative to continuous aerobic training even though it did not show an additional benefit for the three body-composition outcomes. Reviews in adolescents describe HIIT as a time-efficient approach that can improve cardiorespiratory fitness while emphasizing variability in protocols and evidence quality (36). A small study in adolescents with obesity also showed that high-intensity interval exercise could be implemented, although its design and sample size limit comparative conclusions (37). In a school-based randomized trial, HIIT and MICT produced similar body-composition changes, but cardiorespiratory fitness improved more with HIIT (29). A broader pediatric meta-analysis likewise found clearer effects on fitness and selected cardiometabolic markers than on BMI or body fat percentage (38). Exercise selection should therefore reflect the intended outcome, participant preference, functional capacity, safety, supervision, facilities, and likely adherence rather than an assumed weight-loss advantage.

This review has several strengths. Trial eligibility and extracted outcomes were rechecked against original reports, effect directions were harmonized, and random-effects models used REML estimation with modified Hartung–Knapp inference. Robustness was examined through leave-one-out analysis, exclusion of reconstructed effects, inclusion of a study with unclear allocation, subgroup analysis, and exploratory meta-regression; risk of bias was assessed with RoB 2. Three limitations are important. First, the small number of trials and participants limited the precision and power of subgroup analyses, meta-regression, Egger tests, and assessment of uncommon harms. Second, variation in exercise protocols, training dose, co-interventions, and body-fat measurement methods constrained interpretation of the heterogeneous body fat result. Third, 13 trials raised some risk-of-bias concerns, mainly because randomization procedures were incompletely reported or outcome data were missing, and evidence on long-term maintenance, adherence, and adverse events was sparse.

Future trials should use adequately concealed randomization, prospectively registered protocols, sufficient sample sizes, and reproducible descriptions of both HIIT and continuous aerobic training. Standardized body-composition methods, outcome-specific analyzed sample sizes, and reporting of adherence, adverse events, and longer-term follow-up would improve comparability. Prespecified analyses by sex and pubertal stage would be more informative than study-level mean age. On the present evidence, both HIIT and continuous aerobic training can be considered within exercise programs for youth with overweight or obesity, but neither can be regarded as generally more effective for reducing body weight, BMI, or body fat percentage.

## 5 Conclusion

This systematic review and meta-analysis of evidence identified through December 31, 2024 found no statistically significant advantage of high-intensity interval-based training over continuous aerobic training for reducing body weight, BMI, or body fat percentage in youth with overweight or obesity. These findings do not establish equivalence between the two approaches, but they indicate that HIIT may be used as an alternative when it better fits participant preferences, available time, exercise capacity, safety considerations, and implementation conditions. Larger randomized trials with transparent methods, standardized intervention reporting, and longer follow-up are needed to clarify comparative effects and their durability.

## Data Availability

The study-level data extraction sheets, analytic datasets, and statistical code supporting the findings of this study are available from the corresponding author upon reasonable request.

## Supplementary material

Supplementary Table S1 Study-level risk-of-bias judgments for included randomized trials using the Cochrane RoB 2 tool.

Supplementary Table S2 Subgroup analyses of body-composition outcomes by weekly training frequency, intervention duration, and mean participant age.
