# Supplementary material for: Effects of high-intensity interval training versus continuous aerobic training on body weight, body mass index, and body fat percentage in youth with overweight or obesity: a systematic review and meta-analysis
Source: Front Pediatr. 2026 Sep 25;14:1678900. doi: 10.3389/fped.2026.1678900 (PMC13649959; doi:10.3389/fped.2026.1678900)
Supplement: Supplementary file 1 [file Supplementaryfile1.docx]

Supplementary Table S1. Study-level risk-of-bias judgments for included randomized trials using the Cochrane RoB 2 tool

| **Study** | **D1 Randomization** | **D2 Deviations** | **D3 Missing data** | **D4 Measurement** | **D5 Reporting** | **Overall judgment** |
| --- | --- | --- | --- | --- | --- | --- |
| Fernandez et al. 2004 | Some concerns | Low risk | Low risk | Low risk | Low risk | Some concerns |
| Corte de Araujo et al. 2012 | Some concerns | Low risk | Some concerns | Low risk | Low risk | Some concerns |
| Koubaa et al. 2013 | Some concerns | Low risk | Low risk | Low risk | Low risk | Some concerns |
| Starkoff et al. 2014 | Some concerns | Low risk | Some concerns | Low risk | Low risk | Some concerns |
| Kargarfard et al. 2016 | Some concerns | Low risk | Low risk | Low risk | Low risk | Some concerns |
| Lazzer et al. 2017 | Some concerns | Low risk | Low risk | Low risk | Low risk | Some concerns |
| Dias et al. 2018 | Low risk | Low risk | Some concerns | Low risk | Low risk | Some concerns |
| Liang & Hao 2018 | Some concerns | Low risk | Low risk | Low risk | Low risk | Some concerns |
| Miguet et al. 2020 | Some concerns | Low risk | Some concerns | Low risk | Low risk | Some concerns |
| Paahoo et al. 2020 | Some concerns | Low risk | Low risk | Low risk | Low risk | Some concerns |
| Cao et al. 2022 | Low risk | Low risk | Some concerns | Low risk | Low risk | Some concerns |
| Li et al. 2023 | Low risk | Low risk | Some concerns | Low risk | Low risk | Some concerns |
| Zuo et al. 2023 | Low risk | Low risk | Low risk | Low risk | Low risk | Low risk |
| Su et al. 2024 | Some concerns | Low risk | Low risk | Low risk | Low risk | Some concerns |

Note: RoB 2 judgments apply to body weight, BMI, and body fat percentage. D1, randomization process; D2, deviations from intended interventions; D3, missing outcome data; D4, outcome measurement; D5, selection of the reported result. Overall, one trial was judged to be at low risk of bias, 13 raised some concerns, and none was judged to be at high risk.
