# Supplementary material for: Effects of high-intensity interval training versus continuous aerobic training on body weight, body mass index, and body fat percentage in youth with overweight or obesity: a systematic review and meta-analysis
Source: Front Pediatr. 2026 Sep 25;14:1678900. doi: 10.3389/fped.2026.1678900 (PMC13649959; doi:10.3389/fped.2026.1678900)
Supplement: Supplementary file 2 [file Supplementaryfile2.docx]

Supplementary Table S2. Subgroup analyses of body-composition outcomes by weekly training frequency, intervention duration, and mean participant age

| **Outcome** | **Subgroup factor** | **Category** | **Studies (k)** | **Participants (N)** | **MD (95% CI)** | **I² (%)** | **P for interaction** |
| --- | --- | --- | --- | --- | --- | --- | --- |
| Weight (kg) | Weekly training frequency | ≤3 sessions/week | 12 | 379 | −0.37 (−0.89 to 0.16) | 0.0 | 0.773 |
|  |  | ≥4 sessions/week | 1 | 30 | −1.54 (−9.36 to 6.27) | NE |  |
|  | Intervention duration | <12 weeks | 5 | 161 | −0.45 (−1.24 to 0.35) | 0.0 | 0.638 |
|  |  | ≥12 weeks | 8 | 248 | −0.20 (−1.21 to 0.81) | 0.0 |  |
|  | Mean participant age | <13 years | 7 | 228 | −0.00 (−1.09 to 1.09) | 0.0 | 0.403 |
|  |  | ≥13 years | 5 | 163 | −0.46 (−1.26 to 0.33) | 0.0 |  |
| BMI (kg/m²) | Weekly training frequency | ≤3 sessions/week | 13 | 402 | −0.13 (−0.30 to 0.04) | 0.0 | 0.100 |
|  |  | ≥4 sessions/week | 1 | 30 | −2.31 (−4.71 to 0.08) | NE |  |
|  | Intervention duration | <12 weeks | 5 | 161 | −0.13 (−0.40 to 0.14) | 0.0 | 0.868 |
|  |  | ≥12 weeks | 9 | 271 | −0.17 (−0.53 to 0.18) | 8.2 |  |
|  | Mean participant age | <13 years | 8 | 251 | −0.09 (−0.43 to 0.26) | 0.0 | 0.737 |
|  |  | ≥13 years | 5 | 163 | −0.26 (−0.82 to 0.31) | 24.7 |  |
| Body fat (%) | Weekly training frequency | ≤3 sessions/week | 10 | 301 | −0.12 (−1.16 to 0.91) | 55.5 | 0.197 |
|  |  | ≥4 sessions/week | 1 | 30 | −1.99 (−3.49 to −0.48) | NE |  |
|  | Intervention duration | <12 weeks | 3 | 97 | −0.47 (−5.26 to 4.32) | 70.8 | 0.748 |
|  |  | ≥12 weeks | 8 | 234 | −0.22 (−1.50 to 1.05) | 61.0 |  |
|  | Mean participant age | <13 years | 6 | 194 | 0.45 (−1.03 to 1.93) | 62.1 | 0.067 |
|  |  | ≥13 years | 4 | 119 | −1.41 (−2.97 to 0.15) | 11.0 |  |

Note: Values are pooled mean differences (MDs), calculated as HIIT minus continuous aerobic training; negative values favor HIIT. Random-effects models used restricted maximum likelihood with modified Hartung–Knapp inference. P for interaction was obtained from univariable meta-regression. For single-study subgroups, a normal 95% CI is shown and heterogeneity is not estimable (NE). Age-stratified analyses exclude Liang and Hao (2018), which did not report mean participant age.
